# Antioxidant Phenolic Compounds from Pu-erh Tea

**DOI:** 10.3390/molecules171214037

**Published:** 2012-11-27

**Authors:** Hai Ming Zhang, Cheng Fang Wang, Sheng Min Shen, Gang Li Wang, Peng Liu, Zi Mu Liu, Yong Yan Wang, Shu Shan Du, Zhi Long Liu, Zhi Wei Deng

**Affiliations:** 1 State Key Laboratory of Earth Surface Processes and Resource Ecology, Beijing Normal University, Beijing 100875, China; 2 China Academy of Chinese Medical Sciences, Beijing 100700, China; 3 Qingdao Huaren Pharmaceutical Co., Ltd., Qingdao 266101, China; 4 National Institutes for Food and Drug Control, Beijing 100050, China; 5 Analytical and Testing Center, Beijing Normal University, Beijing 100875, China; 6 Department of Entomology, China Agricultural University, Haidian District, Beijing 100193, China

**Keywords:** Pu-erh tea, DPPH*˙*, ABTS˙^+^, chemical constituent, antioxidant activity

## Abstract

Eight compounds were isolated from the water extract of Pu-erh tea and their structures were elucidated by NMR and MS as gallic acid (**1**), (+)-catechin (**2**), (−)-epicatechin (**3**), (−)-epicatechin-3-*O*-gallate (**4**), (−)-epigallocatechin-3-*O*-gallate (**5**), (−)-epiafzelechin-3-*O*-gallate (**6**), kaempferol (**7**), and quercetin (**8**). Their *in vitro* antioxidant activities were assessed by the DPPH and ABTS scavenging methods with microplate assays. The relative order of DPPH scavenging capacity for these compounds was compound **8** > compound **7** > compound **1** > compound **6** > compound **4** ≈ compound **5** > compound **2** > VC (reference) > compound **3**, and that of ABTS scavenging capacity was compound **1** > compound **2** > compound **7** ≈ compound **8** > compound **6** > compound **5** > compound **4** > VC (reference) > compound **3**. The results showed that these phenolic compounds contributed to the antioxidant activity of Pu-erh tea.

## 1. Introduction

Pu-erh tea is a kind of special post-fermented tea, originally produced in the Yunnan province of China for about 1,700 years. Pu-erh tea is obtained by first parching crude green tea leaves (*Camellia sinensis* var. *assamica* (L.) Family: Theaceae) and then it undergoes a secondary fermentation with microorganisms such as *Aspergillus* sp. (postfermented) [1], resulting in a unique type of tea. It was recorded in the Compendium of Materia Medica that Pu-erh tea can expel wind-evil, clear away heat and aid in losing weight [2]. Isolation of some flavonols and catechins from the raw material (the crude green tea) of Pu-erh tea was reported by the Zhou group [3,4]. However, the chemical constituents of Pu-erh tea are thus far not known.

Regarding the functional properties of Pu-erh tea, Sano [5] noted that Pu-erh tea significantly reduced the levels of plasma cholesterol ester and triglyceride in the plasma in rats. In addition, Duh [6] expressed that epicatechin (EC), ascorbic acid, and polyphenolic compounds are present in water extracts of Pu-erh tea (WEPT), which could contribute to the protective effect on oxidative damage as well as nitric oxide scavenging. In an earlier screening of several teas for antioxidant activity showed that Pu-erh tea was a good source of natural antioxidants. Some Pu-erh tea extracts showed a dose dependent scavenging of model free radicals such as the DPPH, superoxide, and nitrogen dioxide radicals [7]. Although Pu-erh tea has demonstrated the potential biological effects mentioned above, there were no reports on the antioxidant activities of the compounds isolated from Pu-erh tea.

The aim of this research was thus to examine the antioxidant properties of phenolic compounds isolated from Pu-erh tea by using two complementary test systems, namely the DPPH free radical-scavenging and ABTS radical-scavenging assays.

## 2. Results and Discussion

### 2.1. Elucidation of the Purified Compounds

**Figure 1:**
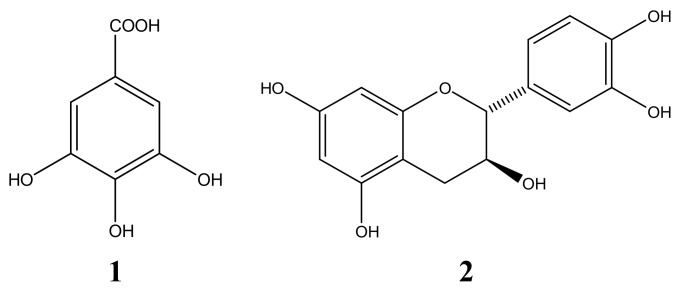

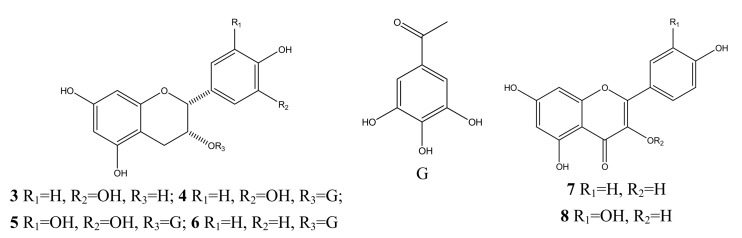
The structures of the compounds isolated from Pu-erh tea.

In this study eight phenolic compounds were isolated from the water extract of Pu-erh tea and their structures were elucidated by NMR and MS as gallic acid (**1**), (+)-catechin (**2**), (−)-epicatechin (**3**), (−)-epicatechin-3-O-gallate (**4**), (−)-epigallocatechin-3-O-gallate (**5**), (−)-epiafzelechin-3-O-gallate (**6**), kaempferol (**7**), and quercetin (**8**), whose structures are shown in Figure 1. It was reported that the main constituents of crude green tea are polyphenols, and the main differences in chemical constituents between Pu-erh tea and crude green tea were the contents of the main catechins [8].

### 2.2. Antioxidant Activity

In a previous study, Guo *et al.* [9] found that the scavenging effects of galloylated catechins (compounds **4** and **5**) on four free radicals were stronger than those of non-galloylated catechins (compounds **2** and **3**), and that the scavenging abilities of compound **2** was stronger than that of its corresponding epimers (compound **3**). The differences between its stereo structures played a more important role in their abilities to scavenge large free radicals. Yokozawa *et al.* [10] also found compounds **4** and **5** had higher antioxidative activity than compounds **2** and **3**, respectively, suggesting that the O-dihydroxy structure in the B ring and the galloyl groups are important determinants for radical scavenging and antioxidative potential. Moreover, Hashimoto *et al.* [11] further confirmed that compound **6** showed the highest activity among the flavan-3-ols was due to not only the presence of a galloyl group, but also to the concomitant contribution of a galloyl group and to the A- and B-rings of a flavan skeleton. In this study, the antioxidant activity of phenols (compounds **2**, **3**, **4**, **5** and **6**) is in agreement with the above reported (Table 1 and Table 2). Scavenging of the stable radical with flavonoids (compounds **7** and **8**) was also examined. At the final concentration of 6.25 μg/mL, compounds **7** and **8** exhibited strong free radical scavenging activity, over 50%. Bors *et al.* [12] reported that the O-dihydroxy (catechol) phenyl ring is an important structure for the antioxidant activity of flavonoids, as seen in compound **8**. Burda*et al.*[13] reported that the antioxidant activity depended on the presence of a flavonol structure or free hydroxyl group at the C-4' position. The effect of compound **7** on scavenging the DPPH free radical, which has no O-dihydroxy phenyl ring, would be attributed to the flavonol structure with a free hydroxyl group at the C-4' position. Compound **1**, the known antioxidant was usually employed in the study for comparing the results [14]. The present results confirmed that compound **1** exhibited strong scavenging activities.

In this study, all the isolated compounds were tested in DPPH˙ and ABTS˙^+^ assays (Table 1 and 2) and their radical scavenging activity was compared with that of the well known natural antioxidant vitamin C. Regarding the IC_50_ values, the scavenging effects of compounds **1**–**8** and reference antioxidant on DPPH˙ decreased in the following order: compound **8** > compound **7** > compound **1** > compound **6** > compound **4** ≈ compound **5** > compound **2** > VC > compound **3**. The scavenging effects of compounds **1**–**8** and reference antioxidant on ABTS˙^+^ decreased in the following order: compound **1** > compound **2** > compound **7** ≈ compound **8** > compound **6** > compound 5 > compound **4** > VC > compound **3**. In the two assays, all of the isolated compounds manifested almost the same patterns of activity as in the DPPH˙ and ABTS˙^+^ method, the only difference was that the compound **2** was excellent scavengers against ABTS˙^+^ (Table 2), but showed moderate scavenging activities against DPPH˙ (Table 1). One of the probable causes of this difference was that the kinetic constants of reactions between ABTS˙^+^ and phenolic compounds were generally higher than that for the reactions between DPPH˙ and phenolic compounds. Moreover, different systems used to measure the scavenging abilities toward the two radicals may also affect the values of radical scavenging activities.

In addition, the radical scavenging capacity of compounds **1**, **2**, **4**, **5**, **6**, **7** and **8** at the applied concentration was higher than that of water extract of Pu-erh tea in the two assay methods, probably because the crude extracts usually contain a great number of components, which may possess different antioxidant or in some cases prooxidant activities as well as being neutral in terms of their effects on oxidation and/or radical scavenging processes.

## 3. Experimental

### 3.1. General Procedures and Reagents

^1^H- and ^13^C-NMR spectra were measured in DMSO-*d_6_* on a 500 MHz Bruker AV-500 (Bruker, Karlsruhe, Germany) at 500 MHz and 125 MHz, respectively, and tetramethylsilane (TMS) was used as an internal standard. Column chromatography was carried out with macroporous resin AB-8 (Nankai University Chemistry Company, Tianjin, China), Polyamide (Zhejiang Huangyan Resin Chemical Industry, Taizhou, China), Sephadex LH-20 (Pharmacia Company, Uppsala, Sweden), MCI-gel CHP20P (Mitsubishi Chemical Corporation, Tokyo, Japan), and Toyopearl HW-40F (Tosoh Corporation, Tokyo, Japan). TLC was performed on precoated silica gel GF_254_ plates (Yantai Jiangyou Silica Gel Development Co. Ltd., Yantai, China). 1,1-Diphenyl-2-picrylhydrazyl (DPPH) and 2,2'-azinobis (3-ethylbenzothiazoline-6-sulfonic acid) diammonium salt (ABTS) were purchased from Sigma Chemical Co. (St. Louis, MO, USA). All other chemicals were of analytical reagent grade and used without any further purification.

### 3.2. Plant Materials

The Pu-erh tea samples were collected from JingDong County (23.56° N latitude and 100.22° E longitude), Yunnan province of China in May 2010. The species was identified by Dr. Liu. Q.R. (College of Life Sciences, Beijing Normal University), and the voucher specimens of the Pu-erh tea were deposited at the Herbarium (BNU) of College of Life Sciences, Beijing Normal University.

### 3.3. Extraction and Isolation of Antioxidant Compounds

The air-dried Pu-erh tea (0.7 kg) was minced and extracted three times with H_2_O (3,000 mL) in an ultrasonic bath at room temperature. The extract was concentrated under reduced pressure to obtain a crude residue (60 g), which was dissolved in water (60 mL) and chromatographed on a AB-8 macroporous adsorptive resin column (70 mm in diameter and 250 mm in height) eluting with a gradient of EtOH-H_2_O (0:100, 10:90, 30:70, 50:50, 90:10), the eluates were concentrated under reduced pressure to dryness and five fractions were obtained. The 30% and 50% ethanol eluent were mingled according to their TLC similarity and subjected to column chromatography over polyamide, eluting with EtOH-H_2_O (10:90, 30:70, 50:50, 90:10) in succession. After further repeated column chromatography over MCI-gel CHP20P, Toyopearl HW-40F and Sephadex LH-20, eight compounds (compounds **1**–**8**) were finally obtained.

### 3.4. Spectroscopic Data

*Gallic acid* (**1**). Colorless crystalline solid; ESI-MS *m/z* 169 [M−H]^−^; ^1^H-NMR δ ppm: 6.93 (2H, s, H-2, H-6); ^13^C-NMR δ ppm: 167.8 (C=O), 145.6 (C-3, 5), 138.2 (C-4), 120.8 (C-1), 109.1 (C-2, 6). The ^1^H- and ^13^C-NMR spectral data were consistent with published data [3].

*(+)-Catechin* (**2**). Colorless crystalline solid; ESI-MS *m/z* 307 [M−H]^−^; ^1^H-NMR δ ppm: δ 6.72 (1H, d, *J* = 1.5 Hz, H-2'), 6.69 (1H, d, *J* = 8.1 Hz, H-5'), 6.59 (1H, dd, *J* = 8.1, 1.5 Hz, H-6'), 5.88 (1H, d, *J* = 2.1 Hz, H-8), 5.70 (1H, d, *J* = 2.0 Hz, H-6), 4.48 (1H, d, *J* = 7.5 Hz, H-2), 3.81 (1H, m, H-3), 2.66 (1H, dd, *J* = 16.0, 5.0 Hz, H-4), 2.63 (1H, dd, *J* = 16.0, 5.0 Hz, H-4); ^13^C-NMR δ ppm: 156.7 (C-5), 156.5 (C-7), 155.7 (C-8a), 145.0 (C-3'), 145.0 (C-4'), 131.0 (C-1'), 118.9 (C-6'), 115.4 (C-5'), 114.8 (C-2'), 99.5 (C-4a), 95.4 (C-6), 94.2 (C-8), 81.3 (C-2), 66.6 (C-3), 29.9 (C-4). The above data were consistent with the literature data [15].

*(−)-Epicatechin* (**3**). Colorless crystalline solid; ESI-MS *m/z* 289 [M−H]^−^; ^1^H-NMR δ ppm: δ 6.88 (1H, d, *J* = 1.2 Hz, H-2'), 6.67 (1H, d, *J* = 8.1 Hz, H-5'), 6.66 (1H, dd, *J* = 8.1, 1.2 Hz, H-6'), 5.89 (1H, d, *J* = 2.1 Hz, H-8), 5.73 (1H, d, *J* = 2.1Hz, H-6), 4.73 (1H, s, H-2), 4.01 (1H, m, H-3), 2.67(1H, dd, *J* = 16.5, 4.5 Hz, H-4), 2.47 (1H, dd, *J* = 16.5, 4.5 Hz, H-4);^13^C-NMR δ ppm: 156.8 (C-5), 156.5 (C-7), 156.2 (C-8a), 144.7 (C-3'), 144.6 (C-4'), 131.1 (C-1'), 118.5 (C-6'), 115.1 (C-2', 5'), 99.0 (C-4a), 95.3 (C-6), 94.5 (C-8), 78.4 (C-2), 65.2 (C-3), 28.6 (C-4). Its NMR data were in accord with the reported data [3].

*(−)-Epicatechin-3-O-gallate* (**4**). Pale brown amorphous powder; ESI-MS *m/z* 441 [M−H]^−^; ^1^H-NMR δ ppm: 6.86 (1H, s, H-2'), 6.83 (2H, s, H-2'', H-6''), 6.75 (1H, d, *J* = 8.0Hz, H-5'), 6.66 (1H, dd, *J* = 8.0, 1.0 Hz, H-6'), 5.94 (1H, d, *J* = 0.6 Hz, H-8), 5.84 (1H, d, *J* = 0.6Hz, H-6), 5.35 (1H, br.s, H-3), 5.04 (1H, s, H-2), 2.94 (1H, dd, *J* = 16.0, 4.5 Hz, H-4), 2.68 (1H, dd, *J* = 16.0, 4.5 Hz, H-4); ^13^C-NMR δ ppm: 165.6 (G,C=O), 119.7 (G-1), 109.0 (G-2, 6), 145.8 (G-3, 5), 138.9 (G-4), 76.9 (C-2), 68.6 (C-3), 26.1 (C-4), 156.1 (C-5), 95.9 (C-6), 156.9 (C-7), 94.8 (C-8), 156.1 (C-9), 97.7 (C-10), 130.0 (C-1'), 114.7 (C-2'), 145.8 (C-3'), 145.1 (C-4'), 118.0 (C-5'), 115.5 (C-6'). The above data were identical to the literature data [3].

*(−)-Epigallocatechin-3-O-gallate* (**5**). Pale brown amorphous powder; ESI-MS *m/z* 457 [M−H]^−^; ^1^H-NMR δ ppm: 6.81 (2H, s, H-2'', H-6''), 6.41 (2H, s, H-2', H-6'), 5.92 (1H, d, *J* = 0.8 Hz, H-8), 5.84 (1H, d, *J* = 0.8 Hz, H-6), 5.34 (1H, br.s, H-3), 4.95 (1H, br.s, H-2), 2.94 (1H, dd, *J* = 16.0, 4.5 Hz, H-4), 2.65 (1H, dd, *J* = 16.0, 4.5 Hz, H-4); ^13^C-NMR δ ppm: 165.7 (G, C=O), 119.8 (G-1), 109.1 (G-2, 6), 145.9 (G-3, 5), 138.7 (G-4), 76.9 (C-2), 68.5 (C-3), 26.1 (C-4), 156.8 (C-5), 95.8 (C-6), 156.0 (C-7), 94.7 (C-8), 156.8 (C-9), 97.8 (C-10), 129.2 (C-1'), 105.9 (C-2', 6'), 145.6 (C-3', 5'), 132.5 (C-4'). The above data were identical to the literature data [3].

*(−)Epiafzelechin-3-O-gallate* (**6**). Pale brown amorphous powder; ESI-MS *m/z* 426 [M−H]^−^; ^1^H-NMR δ ppm: 6.83 (2H, s, H-2'', H-6''), 7.29 (2H, d, *J* = 8.5Hz, H-2', H-6'), 6.70 (2H, d, *J* = 8.5Hz, H-3', H-5'), 5.95 (1H, d, *J* = 2.0 Hz, H-8), 5.84 (1H, d, *J* = 2.0 Hz, H-6), 5.32 (1H, s, H-3), 5.11 (1H, s, H-2), 2.94 (1H, dd, *J* = 16.9, 4.1 Hz, H-4), 2.70 (1H, dd, *J* = 16.9, 4.1 Hz, H-4);^13^C-NMR δ ppm: 165.6 (G, C=O), 119.5 (G-1), 108.9 (G-2, 6), 145.8 (G-3, 5), 138.9 (G-4), 77.0 (C-2), 68.7 (C-3), 26.0 (C-4), 156.9 (C-5), 96.0 (C-6), 157.0 (C-7), 94.8 (C-8), 156.1 (C-9), 97.6 (C-10), 129.2 (C-1'), 128.3 (C-2', 6'), 115.1 (C-3', 5'), 157.2 (C-4'). The above data were identical to the literature data [3].

*Kaempferol* (**7**). Yellow amorphous powder; ESI-MS *m/z* 285 [M−H]^−^; ^1^H-NMR δ ppm: 8.05 (2H, d, *J* = 8.4 Hz, H-2', H-6'), 6.94 (2H, d, *J* = 8.4 Hz,H-3', H-5'), 6.45 (1H, s, H-8), 6.20 (1H, s, H-6); ^13^C-NMR δ ppm: 176.3 (C=O, C-4), 164.2 (C-7), 160.9 (C-9), 159.5 (C-10), 156.7 (C-5), 147.2 (C-2), 136.1 (C-3), 130.0 (C-2', 6'), 122.1 (C-1'), 115.9 (C-3', 5'), 103.5 (C-10), 98.6 (C-6), 93.9 (C-8). The above data were identical to the literature data [3].

*Quercetin* (8). Yellow amorphous powder; ESI-MS *m/z* 301 [M−H]^−^; ^1^H-NMR δ ppm: 7.68 (1H, s, H-2'), 7.55 (1H, d, *J* = 8.4 Hz, H-6'), 6.90 (1H, d, *J* = 8.5 Hz, H-5'), 6.42 (1H, s, H-8), 6.20 (1H, s, H-6); ^13^C-NMR δ ppm: 176.2 (C-4), 164.2 (C-7), 161.2 (C-5), 156.6 (C-9), 148.0 (C-4'), 147.2 (C-2),145.4 (C-3'), 136.3 (C-3), 122.4 (C-1'), 120.5 (C-6'), 116.0 (C-2'), 115.5 (C-5'), 103.4 (C-10), 98.5 (C-6), 93.8 (C-8). The above data were identical to the literature data [3].

### 3.5. DPPH˙ Scavenging Activity

DPPH˙ scavenging activity of the compounds isolated from Pu-erh tea was carried out as described by Shimada [16] with minor modifications. Briefly, a 0.1 mM solution of DPPH˙ in 100% MeOH was prepared. One mL of this solution was added to of sample solution in MeOH (4 mL) at different concentrations (12.5–200 μg/mL). The mixture was shaken vigorously and incubated for 15 min in the dark at room temperature until stable absorption values were obtained. The reduction of the DPPH˙ radical was measured by continuously monitoring the decrease in absorption at 517 nm. In the control, MeOH was substituted for samples. The DPPH radical scavenging activity was calculated by the following equation:

Scavenging effect (%) = (1 − A_sample517_/A_control517_) × 100 where A_sample517_ was the absorbance in the presence of the sample, and A_control517_ was the absorbance of the control reaction containing all reagents except the test sample. The median inhibitory concentration (IC_50_, the effective concentration at which 50% of DPPH radicals was scavenged) was calculated using the linear relation between the inhibitory probability and concentration logarithm according to methods outlined by Sakuma [17].

### 3.6. ABTS˙^+^ Scavenging Activity

The radical scavenging activity of the isolated compounds against ABTS˙^+^ was determined according to Re [18]. The ABTS˙^+^ (cation radical) was produced by the reaction between 5 mL of 14 mM ABTS solution and 5 mL of 4.9 mM potassium persulfate (K_2_S_2_O_8_) solution, stored in the dark at room temperature for 16 h. Before use, this solution was diluted with distilled water to get an absorbance of 0.900 ± 0.020 at 734 nm. In a final volume of 1 mL, the reaction mixture comprised 0.8 mL of ABTS˙^+^ solution and 0.2 mL of the sample extract at various concentrations. The decrease in absorbance value was measured at 734 nm after 6 min. The percent scavenging of ABTS˙^+^ (cation radical) was calculated by the following equation:

Scavenging effect (%) = (1 − A_sample734_/A_control734_) × 100 where A _sample734_ was the absorbance in the presence of the sample, and A_control734_ was the absorbance of the control reaction containing all reagents except the test sample. The scavenging ability of the samples was expressed as IC_50_ value, which is the effective concentration at which 50% of ABTS radicals were scavenged. The IC_50_ value was calculated from the scavenging activities (%) versus concentrations of respective sample curve. The median inhibitory concentration (IC_50_, the effective concentration at which 50% of ABTS radicals was scavenged) was calculated using the linear relation between the inhibitory probability and concentration logarithm according to methods outlined by Sakuma [17].

### 3.7. Statistical Analysis

All the tests were performed in triplicate. The results were given as means ± S.D. Analysis of variance and significant differences among means were tested by one-way ANOVA, using SPSS (Version 13.0 for Windows, SPSS Inc., Chicago, IL, USA). When significant main effects existed, differences were tested by a multiple comparison Tukey test at 95% confidence. Percentage data were arcsine transformed before statistical analysis to ensure homogeneity of variance.

## 4. Conclusions

Phenolic components of Pu-erh tea were studied for the first time. Eight compounds were isolated and their antioxidant activities were evaluated using two microplate assay methods. The antioxidative activities of compounds **1**, **2**, **4**, **5**, **6**, **7** and **8** were higher than that of vitamin C, suggesting the tea could be a good source of natural antioxidants.

## Figures and Tables

**1**, gallic acid; **2**, (+)-catechin; **3**, (−)-epicatechin; **4**, (−)-epicatechin-3-O-gallate; **5**, (−)-epigallocatechin-3-O-gallate; **6**, (−)-epiafzelechin-3-O-gallate; **7**, kaempferol; **8**, quercetin.

**Table 1:** DPPH radical scavenging rate.

Investigated materials	DPPH radical scavenging rate (%)						IC_50_ (μg/mL)
	200 (μg/mL)	100 (μg/mL)	50 (μg/mL)	25 (μg/mL)	12.50 (μg/mL)	6.25 (μg/mL)	
Water extract	66.75 ± 1.96 ^b^	63.87 ± 1.92 ^b^	58.30 ± 2.00 ^b^	54.17 ± 1.57 ^d^	46.53 ± 1.26 ^c^	29.15 ± 0.94 ^d^	25.79
**1**	82.92 ± 2.58 ^a^	82.11 ± 3.01 ^a^	82.39 ± 3.58 ^a^	80.29 ± 3.41 ^a,b,c^	60.29 ± 1.70 ^a^	50.90 ± 1.70 ^b^	4.98
**2**	68.10 ± 2.30 ^b^	67.21 ± 2.08 ^b^	60.55 ± 2.61 ^b^	57.56 ± 2.41 ^d^	51.38 ± 1.55 ^b^	50.55 ± 1.44 ^b^	7.22
**3**	69.57 ± 2.25 ^b^	53.09 ± 1.60 ^c^	29.74 ± 1.11 ^c^	23.32 ± 1.08 ^e^	12.83 ± 0.46 ^d^	10.94 ± 0.28 ^e^	92.43
**4**	82.61 ± 3.14 ^a^	82.72 ± 2.96 ^a^	78.01 ± 2.60 ^a^	73.44 ± 2.30 ^c^	59.78 ± 2.08 ^a^	45.82 ± 1.12 ^c^	6.90
**5**	83.36 ± 2.40 ^a^	83.57 ± 2.87 ^a^	79.41 ± 3.95 ^a^	76.50 ± 2.59 ^b,c^	58.36 ± 2.14 ^a^	45.98 ± 1.40 ^c^	6.84
**6**	84.07 ± 1.79 ^a^	84.07 ± 3.28 ^a^	79.34 ± 3.65 ^a^	75.81 ± 3.07 ^b,c^	59.48 ± 2.30 ^a^	46.96 ± 1.07 ^c^	6.61
**7**	84.08 ± 3.00 ^a^	84.03 ± 2.61 ^a^	83.37 ± 3.38 ^a^	83.49 ± 3.16 ^a,b^	60.48 ± 1.79 ^a^	52.90 ± 1.44 ^b^	4.64
**8**	83.92 ± 1.87 ^a^	83.44 ± 2.53 ^a^	83.46 ± 3.34 ^a^	83.18 ± 3.02 ^a,b^	63.31 ± 2.79 ^a^	59.14 ± 1.13 ^a^	2.79
Vitamin C	84.72 ± 2.55 ^a^	83.83 ± 2.66 ^a^	85.20 ± 3.16 ^a^	85.40 ± 3.46 ^a^	51.10 ± 2.01 ^b^	45.61 ± 1.25 ^c^	7.50

Data expressed as means ± standard deviation. Means in the same column followed by same letters do not differ significantly (*p* > 0.05) in ANOVA test. IC_50_ values were calculated based only on five concentrations (6.25–100 μg/mL).

**Table 2:** ABTS radical scavenging rate.

Investigated materials	ABTS radical scavenging rate (%)						IC_50_ (μg/mL)
	200 (μg/mL)	100 (μg/mL)	50 (μg/mL)	25 (μg/mL)	12.5 (μg/mL)	6.25 (μg/mL)	
Water extract	71.34 ± 2.29 ^b^	58.72 ± 1.50 ^b^	54.94 ± 2.06 ^d^	51.23 ± 2.08 ^d^	42.07 ± 1.79 ^d^	27.54 ± 0.99 ^e^	32.30
**1**	95.45 ± 3.46 ^a^	95.59 ± 3.51 ^a^	94.48 ± 3.29 ^a^	94.31 ± 3.38 ^a^	83.43 ± 3.34 ^a^	60.12 ± 1.76 ^a^	2.97
**2**	94.24 ± 1.93 ^a^	94.40 ± 3.09 ^a^	93.38 ± 3.38 ^a,b^	93.33 ± 3.22 ^a^	78.15 ±2 .12 ^a^	60.11 ± 2.07 ^a^	3.12
**3**	69.75 ± 2.13 ^b^	51.77 ± 2.00 ^b^	27.74 ± 1.11 ^e^	24.58 ± 0.77 ^e^	15.45 ± 0.51 ^e^	10.08 ± 0.31^f^	95.14
**4**	95.57 ± 2.89 ^a^	94.32 ± 3.03 ^a^	86.25 ± 2.55 ^b,c^	70.12 ± 1.93 ^c^	66.22 ± 2.00 ^b^	46.98 ± 1.58 ^d^	7.13
**5**	95.05 ± 3.91 ^a^	93.22 ± 2.77 ^a^	78.53 ± 2.39 ^c^	71.27 ± 2.18 ^c^	67.24 ± 2.03 ^b^	47.51 ± 1.29 ^d^	6.72
**6**	95.16 ± 4.04 ^a^	95.35 ± 3.34 ^a^	90.65 ± 2.83 ^a,b^	85.20 ± 2.95 ^b^	68.24 ± 1.93 ^b^	47.33 ± 1.30 ^d^	6.31
**7**	95.14 ± 4.18 ^a^	95.01 ± 3.85 ^a^	95.53 ± 3.29 ^a^	90.54 ± 2.79 ^a,b^	83.23 ± 2.40 ^a^	52.64 ± 1.38 ^b,c^	4.09
**8**	95.21 ± 2.81^a^	94.38 ± 3.24 ^a^	93.66 ± 2.74 ^a,b^	90.77 ± 2.98 ^a,b^	80.61 ± 2.88 ^a^	53.33 ± 1.28 ^b^	4.04
Vitamin C	95.32 ± 3.15 ^a^	95.48 ± 3.45 ^a^	95.31 ± 2.92 ^a^	95.03 ± 2.97 ^a^	53.39 ± 1.61 ^c^	49.20 ± 1.37 ^c,d^	7.20

Data expressed as means ± standard deviation. Means in the same column followed by same letters do not differ significantly (*p* > 0.05) in ANOVA test. IC_50_ values were calculated based only on five concentrations (6.25–100 μg/mL).

## References

[1] Jeng K.C., Chen C.S., Fang Y.P., Hou R.C., Chen Y.S. (2007). Effect of microbial fermentation on content of statin, GABA, and polyphenols in Pu-erh tea. J. Agric. Food Chem..

[2] Wang D., Xu K.L., Zhong Y., Luo X., Xiao R., Hou Y. (2011). Acute and subchronic oral toxicities of Pu-erh black tea extract in Sprague-Dawley rats. J. Ethnopharmacol..

[3] Zhou Z.H., Yang C.R. (2000). Chemical constituents of crude green tea, the material of Pu-erh tea in Yunnan. Acta Bot. Yunnanica.

[4] Zhou Z.-H., Zhang Y.-J., Xu M., Yang C.-R. (2005). Puerins A and B, two new 8-C substituted flavan-3-ols from Pu-erh tea. J. Agric. Food Chem..

[5] Sano M., Takeuaka Y., Kojima R., Saito S.I., Tomita I., Katou M., Shibuya S. (1986). Effects of Pu-erh tea on lipid metabolism in rats. Chem. Pharm. Bull..

[6] Duh P.D., Yen G.C., Yen W.J., Wang B.S., Chang L.W. (2004). Effects of Pu-erh tea on oxidative damage and nitric oxide scavenging. J. Agric. Food Chem..

[7] Gong J.S., Peng C.X., He X., Li J.H., Li B.C., Zhou H.J. (2009). Antioxidant activity of extracts of Pu-erh tea and its material. Asian J. Agric. Sci..

[8] Qian Z.M., Guan J., Yang F.Q., Li S.P. (2011). Identification and quantification of free radical scavengers in Pu-erh tea by HPLC-DAD-MS coupled online with 2,2'-azinobis(3-ethylbenzthiazolinesulfonic acid) diammonium salt assay. J. Agric. Food Chem..

[9] Guo Q., Zhao B.L., Hou J.W. (1999). ESR study on the structure-antioxidant activity relationship of tea catechins and their epimers. Biochim. Biophys. Acta.

[10] Yokozawa T., Cho E.J., Hara Y., Kitani K. (2000). Antioxidative activity of green tea treated with radical initiator 2,2'-azobis(2-amidinopropane) dihydrochloride. J. Agric. Food Chem..

[11] Hashimoto F., Ono M., Masuoka C., Ito Y., Sakata Y., Shimizu K., Nonaka G., Nishioka I., Nohara T. (2003). Evaluation of the anti-oxidative effect (*in vitro*) of tea polyphenols. Biosci. Biotechnol. Biochem..

[12] Bors W., Heller W., Michel C., Saran M. (1990). Flavonoids as antioxidants: Determination of radical-scavenging efficiencies. Methods Enzymol..

[13] Burda S., Oleszek W. (2001). Antioxidant and antiradical activities of flavonoids. J. Agric. Food Chem..

[14] Ahmad M.R., Sastry V.G., Bano N. (2011). Antibacterial and antioxidant activities of some novel chalcone derivatives. J. Pharm. Res..

[15] Zou Y.L., Dong B.S., Zhang F.Q., He M., Li C., Ou L.C., He Y.P. (2009). Chemical Constituents of Pu-erh tea. Yunnan Chem. Technol..

[16] Shimada K., Fujikawa K., Yahara K., Nakamura T. (1992). Antioxidative properties of xanthan on the autoxidation of soybean oil in cyclodextrin emulsion. J. Agric. Food Chem..

[17] Sakuma M. (1998). Probit analysis of preference data. Appl. Entomol. Zool..

[18] Re R., Pellegrini N., Proteggente A., Pannala A., Yang M., Rice-Evans C. (1999). Antioxidant activity applying an improved ABTS radical cation decolorization assay. Free Radic. Biol. Med..

